# Profibrotic epithelial phenotype: a central role for MRTF and TAZ

**DOI:** 10.1038/s41598-019-40764-7

**Published:** 2019-03-13

**Authors:** Janne Folke Bialik, Mei Ding, Pam Speight, Qinghong Dan, Maria Zena Miranda, Caterina Di Ciano-Oliveira, Michael M. Kofler, Ori D. Rotstein, Stine F. Pedersen, Katalin Szászi, András Kapus

**Affiliations:** 1 Keenan Research Centre for Biomedical Science of the St. Michael’s Hospital, University of Toronto, Toronto, ON M5B 1T8 Canada; 2 Dept. Surgery, University of Toronto, Toronto, ON M5B 1T8 Canada; 3 Dept. Biochemistry, University of Toronto, Toronto, ON M5B 1T8 Canada; 4 Dept. Cell and Developmental Biology, University of Copenhagen, Copenhagen, DK-2100 Denmark

## Abstract

Epithelial injury is a key initiator of fibrosis but - in contrast to the previous paradigm - the epithelium *in situ* does not undergo wide-spread epithelial-mesenchymal/myofibroblast transition (EMT/EMyT). Instead, it assumes a Profibrotic Epithelial Phenotype (PEP) characterized by fibrogenic cytokine production. The transcriptional mechanisms underlying PEP are undefined. As we have shown that two RhoA/cytoskeleton-regulated transcriptional coactivators, Myocardin-related transcription factor (MRTF) and TAZ, are indispensable for EMyT, we asked if they might mediate PEP as well. Here we show that mechanical stress (cyclic stretch) increased the expression of transforming growth factor-β1 (TGFβ1), connective tissue growth factor (CTGF), platelet-derived growth factor and Indian Hedgehog mRNA in LLC-PK1 tubular cells. These responses were mitigated by siRNA-mediated silencing or pharmacological inhibition of MRTF (CCG-1423) or TAZ (verteporfin). RhoA inhibition exerted similar effects. Unilateral ureteral obstruction, a murine model of mechanically-triggered kidney fibrosis, induced tubular RhoA activation along with overexpression/nuclear accumulation of MRTF and TAZ, and increased transcription of the above-mentioned cytokines. Laser capture microdissection revealed TAZ, TGFβ1 and CTGF induction specifically in the tubular epithelium. CCG-1423 suppressed total renal and tubular expression of these proteins. Thus, MRTF regulates epithelial TAZ expression, and both MRTF and TAZ are critical mediators of PEP-related epithelial cytokine production.

## Introduction

Chronic kidney disease (CKD), affecting 12% of the North-American population is considered an “epidemic”^1^. Renal fibrosis, characterized by excessive extracellular matrix deposition and consequent disruption of the kidney architecture is the final common pathogenic mechanism through which CKD, independent of etiology, progresses toward end-stage kidney disease^2–5^. Ever since the myofibroblast was recognized as the central cellular mediator of fibrogenesis^6^, understanding the origins and activation of this mesenchymal cell type have become the focus of fibrosis research^7,8^. This scenario poses a mechanistic challenge. There is general consensus that epithelial injury (due to inflammatory, hypoxic, metabolic or obstructive/mechanical insults) is an essential triggering factor in tubulointerstitial fibrosis^9–11^. In fact, epithelium-targeted expression of damage molecules (e.g. cell cycle inhibitors, cholera toxin) is sufficient to provoke profound fibrosis^12,13^. On the other hand, the executors of fibrotic tissue remodeling are mesenchymal cells, including fibroblasts and their contractile, α-smooth muscle actin (αSMA)-expressing counterparts, the myofibroblasts. What is the link then between epithelial initiation and mesenchymal execution?

The previous paradigm attributed a *direct role* to the tubular epithelium in (myo)fibroblast generation via epithelial-mesenchymal transition. This notion was based on histological “snapshots” showing the co-existence of epithelial and mesenchymal markers in the injured epithelium *in vivo* (reviewed in^14–16^) and the undebated capacity of tubular cells to transition to myofibroblasts *in vitro*^17,18^. Early fate-tracing experiments proposed that nearly 40% of myofibroblasts might originate from tubular cells^19^. However, subsequent lineage tracking studies challenged this view, claiming that EMT plays no role in fibrogenesis^20–22^. A recent analysis provided a more nuanced view, proposing that although full EMT occurs during fibrogenesis, it is a rare event accounting for only a small portion (<5%) of myofibroblasts^23^. Accordingly, an alternative paradigm is emerging, which assigns a crucial but *indirect role* to the epithelium in fibrogenesis. Upon injury, tubular cells attain an activated state, which we term “*profibrotic epithelial phenotype*” or PEP. This may be regarded as a partial EMT^24,25^, which does not go “all the way” to fibroblast/myofibroblast transition. Instead, its major characteristic is a secretory state. Indeed, the epithelium has been shown to be the source of potent fibrogenic mediators including transforming growth factor β (TGFβ1), connective tissue growth factor (CTGF), Platelet-derived Growth Factor (PDGF), and Indian Hedgehog (IHH)^12,26–29^. These in turn activate the neighboring mesenchymal cells, promoting fibroblast/pericyte proliferation and migration, extracellular matrix (ECM deposition and myofibroblast transition^10^. However, while PEP may be crucial for fibrogenesis, the signaling/transcriptional mechanisms underlying the evolution of this state remain poorly understood.

Previous work in our lab has focused on molecular mechanisms underlying transcriptional reprogramming during full-blown epithelial-myofibroblast transition (EMyT), characterized by α-SMA expression. We have shown that an intact tubular epithelium is highly resistant to myofibroblast transition, inasmuch that TGFβ1, the most potent fibrogenic and EMT-inducing cytokine is insufficient, in itself, to provoke EMyT^30–32^. The required second hit is increased contractility and cytoskeletal remodeling, which can be induced by cell contact injury or mechanical stress^30–35^. Searching for the molecular mechanisms, we have shown that two Rho GTPase/cytoskeleton-regulated transcriptional coactivators, myocardin-related transcription factor (MRTF) and transcriptional co-activator with PDZ-binding motif (TAZ) are indispensable for EMyT^31,32,34,35^. Both MRTF and TAZ are central mediators in mechanotransduction^36,37^, regulated at the level of their nucleo-cytoplasmic shuttling. Increased actin polymerization, myosin phosphorylation (contractility) or loss of cell contacts, which are well-known features of epithelial injury, have all been shown to facilitate nuclear accumulation of MRTF^31,32,38–40^ and TAZ^31,41,42^. In the nucleus, MRTF interacts with serum response factor (SRF) and this complex drives gene expression via the CC(A/T)_6_GG cis- element, the CArG-box^43^. TAZ (and its paralog YAP), which in addition to their regulation by the cytoskeleton are also controlled by the Hippo kinase pathway^44^, activate TEADs and other transcription factors in the nucleus^45^. Furthermore, we and others have shown that MRTF and TAZ engage in a multilevel crosstalk with each other and with elements of TGFβ1 signaling (Smad3)^35,46,47^. We found that MRTF can regulate TAZ expression and that TAZ interacts with MRTF and Smad3, and primes the epithelium for TGFβ1-induced transformation^34,35,48^. Moreover, both MRTF^49,50^ and TAZ^51–53^ have emerged as mediators of organ fibrosis.

Given the essential roles of MRTF and TAZ in EMyT, we asked if these factors may also be mediators of PEP, the chief characteristic of which is epithelial cytokine production. To address this key question, we used two approaches: we assessed *in vitro* whether interference with MRTF or TAZ signaling alters mechanically-induced cytokine production in tubular cells. Further, using unilateral ureteral obstruction (UUO), a mechanical model of renal fibrosis, we tested whether pharmacological inhibition of MRTF alters fibrogenic TAZ and cytokine expression in the whole kidney and the tubular epithelium. Our data suggest that MRTF and TAZ are important mediators of PEP and therefore are promising pharmacological targets to interfere with changes in epithelial plasticity, a key driver of fibrogenesis.

## Results

### Inhibition of MRTF, TAZ or RhoA strongly suppresses mechanical stress-induced fibrogenic cytokine expression in epithelial cells

**Figure 1:**
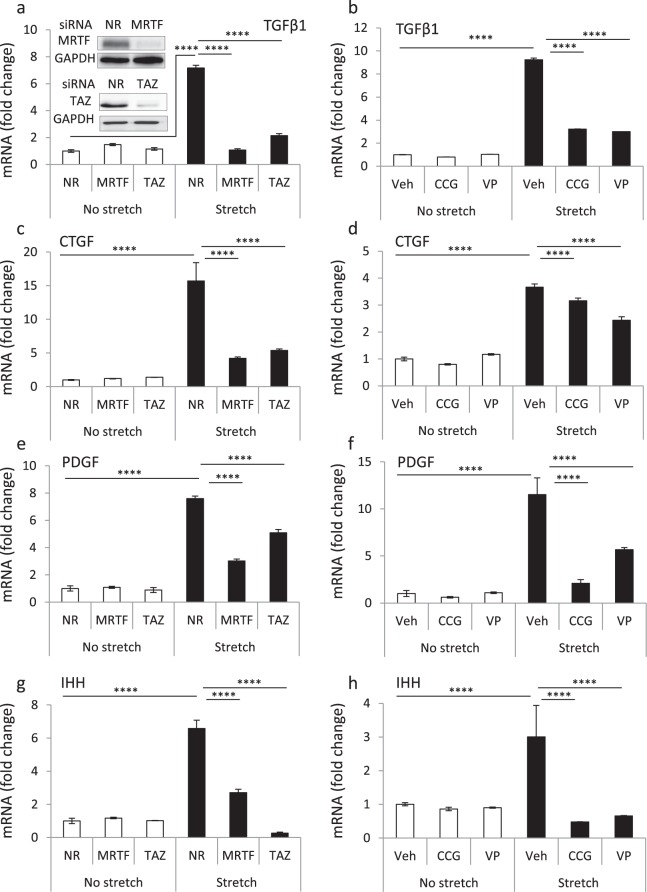
Mechanical stress induces the expression of fibrogenic cytokines in tubular cells via MRTF- and TAZ-dependent mechanisms. LLC-PK1 cells were grown to confluence on Flexcell membranes and transfected with non-related (NR), MRTF-A + B- (50 nM each) or TAZ-specific (50 nM) siRNA for 48 h (**a**,**c**,**e**,**g**), or serum-deprived overnight and pretreated with vehicle (Veh), CCG-1423 (CCG, 5 μM) or verteporfin (VP, 1 μM) for 30 min (**b**,**d**,**f**,**h**), as shown. Cells were then left unchallenged (No stretch) or subjected to cyclic stretch (Stretch, 10% elongation at 1 Hz) for 3 h. The pharmacological inhibitors were present throughout the course of the experiment. Cells were lysed, total cellular mRNA was isolated and reverse-transcribed and qPCR was performed using primers targeting TGFβ1 (**a**,**b**), CTGF (**c**,**d**), PDGF-B (**e**,**f**) and IHH (**g**,**h**). The expression of cytokine mRNAs was normalized to GAPDH in the same samples, and data is presented as fold changes compared to the untreated and unchallenged samples (n = 3, in duplicate). To assess residual activation after inhibitory interventions, statistical analysis was also performed between no-stretch + inhibitor and stretch + inhibitor conditions. TGFβ: non-stretch siMRTF and siTAZ vs. stretch siMRTF and siTAZ, p < 0.05 and p < 0.0001; non-stretch CCG and VP vs. stretch CCG and VP, p < 0.01 and p < 0.05. CTGF: non-stretch siMRTF and siTAZ vs. stretch siMRTF and siTAZ, ns and p < 0.01; non-stretch CCG and VP vs. stretch CCG and VP, p < 0.0001 and p < 0.0001. PDGF: non-stretch siMRTF and siTAZ vs. stretch siMRTF and siTAZ, p < 0.0001 and p < 0.0001; non-stretch CCG and VP vs. stretch CCG and VP, ns and p < 0.001. IHH: non-stretch siMRTF and siTAZ vs. stretch siMRTF and siTAZ, p < 0.0001 and ns; non-stretch CCG and VP vs. stretch CCG and VP, ns and ns. The representative Western blots (inset in panel a) document the downregulation of MRTF and TAZ by the corresponding siRNAs.

Initially we tested whether mechanical stress, a relevant inducer of fibrosis^54,55^, alters fibrogenic cytokine expression in kidney tubular cells, and if so whether such responses might be dependent on MRTF and/or TAZ signaling. Since cyclic stretch is an adequate mimic of pathophysiologic tubular mechanostress^56,57^, and we have previously shown that it induces robust MRTF and TAZ translocation in LLC-PK1 proximal tubular cells^35^, we used this stimulus for these proof-of-principle experiments. The applied 3-hour stretch regimen significantly increased mRNA levels of all of the examined fibrogenic cytokines, namely TGFβ1, CTGF, PDGF-B, and IHH (Fig. 1). To assess the involvement of MRTF and/or TAZ, we downregulated these molecules by the respective siRNAs. Similar to our previous findings^35,58^ we obtained >80% reduction in the corresponding protein levels (Fig. 1a, inset) (Extended blots for all figures are shown in Supplementary Fig. 1). Importantly, MRTF A + B or TAZ silencing completely abolished or significantly mitigated the stretch-triggered increase in all of the investigated cytokine mRNAs (Fig. 1a,c,e,g). We next tested whether small molecule inhibitors of these pathways, which can also be applied *in vivo*, could also reduce mechanically induced cytokine production. CCG-1423 inhibits MRTF’s nuclear translocation and its binding to SRF^59,60^, whereas verteporfin destabilizes TAZ/YAP and prevents their interaction with TEAD^53,61,62^. Both CCG-1423 and verteporfin significantly mitigated the stretch-induced rise in the mRNA levels of all four cytokines, exerting complete or partial suppression (Fig. 1b,d,f,h).

**Figure 2:**
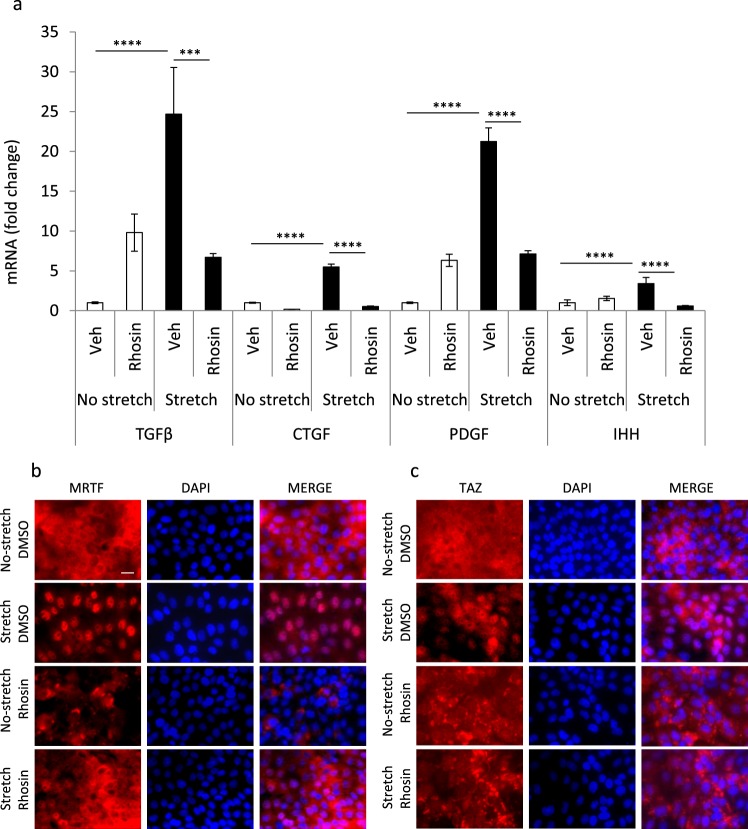
RhoA is a central mediator of mechanostress-induced expression of fibrogenic cytokines and nuclear translocation of MRTF and TAZ. LLC-PK1 cells, grown to confluence on Flexcell membranes, were pretreated with vehicle (DMSO) or the RhoA GTPase inhibitor Rhosin (10 μM) for 30 min and then left unstimulated or exposed to cyclic stretch (10% elongation 1 Hz) for 3 h. (**a**) mRNA levels for the indicated cytokines were determined by qPCR as in Fig. 1 (mean ± S.D, n = 3–6). (**b**). At the end of the experiments, cells on the Flexcell membranes were fixed and processed for immunostaining for MRTF and TAZ, as indicated. DAPI was used as a nuclear marker. Note that Rhosin prevented the stretch-induced increase in the mRNA level of each of the investigated cytokines and inhibited the nuclear translocation of both MRTF and TAZ. Scale bar: 10 μm.

While multiple inputs can activate MRTF and TAZ, RhoA activation and the ensuing actin polymerization are key inputs in the context of mechanotransduction^36^. To test if RhoA activation plays a significant role in mechanically induced fibrogenic cytokine expression, we pretreated LLC-PK1 cells with Rhosin, an inhibitor of the interaction of RhoA with its guanine nucleotide exchange factors^63^. This strategy was chosen because Rhosin did not alter cell viability or the attachment of epithelial cells to the stretchable membranes in these short-term experiments. In non-stretched cells, Rhosin elevated the basal mRNA expression of some cytokines (TGFβ1, PDGF), while it had no effect on others (CTGF, IHH) (Fig. 2a). This finding might be due to a RhoA inhibition-induced shift in the activity of other Rho family small GTPases, which might also impact cytokine expression. More importantly, Rhosin entirely abolished the stretch-induced increase in expression of all four cytokines, resulting in significantly lower cytokine mRNA expression in Rhosin- vs. vehicle-treated stretched cells (Fig. 2a). Accordingly, Rhosin prevented the stretch-provoked nuclear translocation of both MRTF (Fig. 2b) and TAZ (Fig. 2c). Interestingly, Rhosin appeared to promote the accumulation of TAZ in cytosolic puncta (Fig. 2c).

Taken together, these experiments show that mechanical stress induces fibrogenic cytokine expression in renal tubular cells in a RhoA -, MRTF- and TAZ-dependent manner.

### MRTF is a key regulator of TAZ expression and fibrogenic cytokine production in the early phase of renal fibrogenesis

Next, we asked whether MRTF and TAZ are also relevant regulators of cytokine expression during fibrogenesis. To approach this question, we chose an obstructive nephropathy model, UUO because it triggers fibrosis primarily by epithelial stress, exerted by a mechanical factor (high intratubular pressure)^64^. As our goal was to delineate events in the early (epithelial) phase of fibrogenesis, in most experiments we used short-term (3-day) UUO.

**Figure 3:**
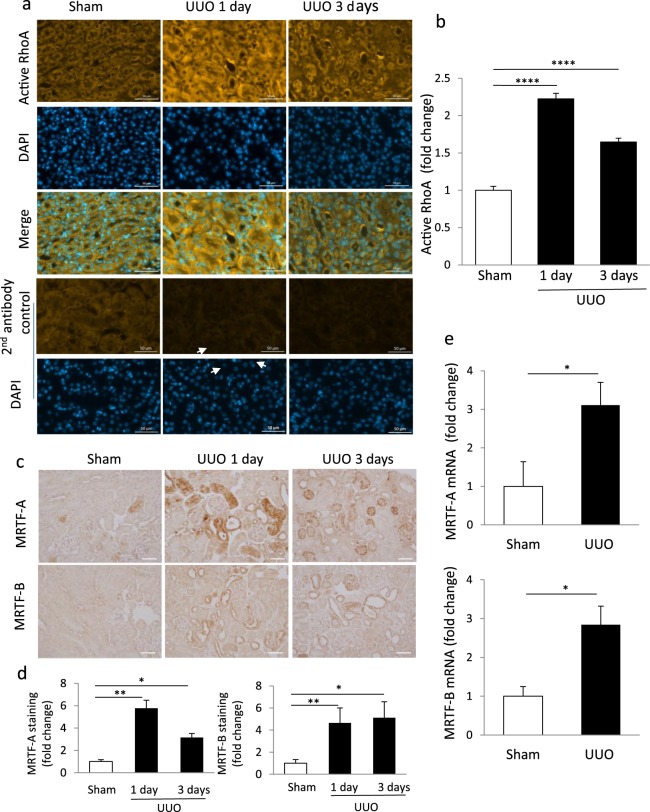
Early UUO provokes RhoA GTPase activation and increases total and nuclear MRTF expression in tubular cells. Mice were sham operated or underwent UUO (1 or 3 days as shown), as described in the Methods. (**a**,**b**) Renal tissue sections were processed for immunohistochemical analysis using a primary antibody specific for active RhoA. Nuclei were stained with DAPI. Bar: 50 μm (**a**). Intensity of the labeling was quantified in >20 selected areas of 100 μm diameter/animal in 2–3 animals in each group, using the Zen software. The intensities were background corrected by subtracting the average labeling obtained in samples exposed to the secondary antibody only. (**c**) Immunohistochemical staining was performed for MRTF-A (upper panels) or MRTF-B (lower panels on kidney section obtained after sham (n = 3) 1-day (n = 4) or 3-day (n = 3) UUO surgery. Bar: 50 µm. (**d**) Staining intensities were quantified as in (**a**) using 5–7 randomly selected fields in 3 sham and 4 UUO animals in each group. (**e**) Whole kidney extracts were prepared from sham (n = 3) or UUO-treated mice (3 days, n = 4) and mRNA levels for MRTF-A (upper panel) and MRTF-B (lower panel) were quantified by qPCR.

We first tested whether UUO alters tubular Rho activity. To this end, control and UUO samples were stained with an activation-specific antibody that selectively binds to the GTP-bound form of RhoA (Fig. 3a). Active RhoA labeling, predominantly in the tubular epithelium, was markedly stronger in 1- and 3-day UUO sections compared to sham samples. Typically the signal was diffuse at day 1, while at day 3 a more pronounced membrane staining was evident in tubular cells. Quantification of full kidney sections revealed significantly elevated levels at both times, the earlier response being stronger (Fig. 3b).

Since various fibrogenic stimuli were shown to induce MRTF expression, activation and nuclear translocation in cultured cells^32,65–67^, we next examined if UUO altered MRTF expression or distribution in kidney samples (Fig. 3c). Immunostaining revealed that UUO provoked a rapid and substantial increase in both MRTF-A and MRTF-B labeling, predominantly in the tubular epithelium (Fig. 3c, and Supplementary Fig. 2a–e). Quantitation showed a several-fold difference compared to the sham levels after both 1- and 3-day UUO (Fig. 3d, Supplementary Fig. 2a–d) and this effect persisted even at day 7 (Supplementary Fig. 2f,g). This rise seemed to proportionally affect the cytosolic and nuclear compartments. Image analysis showed that the nuclear content of MRTF, the driver of MRTF-dependent transcription, was significantly higher in the UUO- treated kidneys than in the sham ones (Supplementary Fig. 2c,e). To verify changes in MRTF expression by an independent method, we performed qPCR; UUO induced a significant rise in both MRTF-A and B mRNA (Fig. 3e and Supplementary Fig. 2h for 7 day UUO).

**Figure 4:**
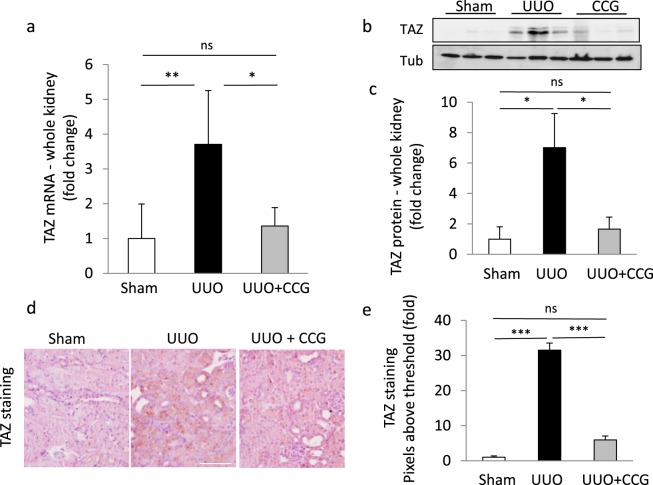
The MRTF inhibitor CCG-1423 suppresses the UUO-induced rise in renal TAZ mRNA and protein expression. (**a**–**c**) Whole kidney extracts were prepared from mice 3 days after sham or UUO surgery and analyzed for TAZ mRNA by qPCR (a) (mean ± S.D., n = 5/per group), or TAZ protein using Western blotting (**b**,**c**). Representative blots are shown from 3 animals (**b**). Protein levels were quantified by densitometry and normalized to tubulin (n = 5/per group) (**c**). (**d**,**e**) TAZ was visualized by immunohistochemical staining in kidney sections from sham, UUO or CCG-1423-treated UUO mice (**d**). Images were quantified by determining the number of pixels above the threshold under the various conditions, and expressed as fold change compared to sham. At least 10 fields were analyzed from 2 randomly selected animals in each group (**e**) (mean ± .S.E., n > 20/group). Bar 200 μm.

Having seen that UUO promoted RhoA/MRTF signaling in the epithelium, we set out to determine the potential role of MRTF in TAZ status and the cytokine responses. To this end, mice were distributed into three treatment groups that underwent sham operation, UUO (3 days) or CCG-1423 treatment plus UUO (3 days). The drug was administered intraperitoneally each day, starting at the day of the surgery. Since our recent studies showed that MRTF promotes TAZ expression *in vitro*^35,48^ and that UUO elevates renal TAZ levels^48^, we tested if CCG-1423 might impact this response. As expected, 3-day UUO significantly increased total renal TAZ mRNA and protein expression, as revealed by qPCR, Western blotting and quantified immunohistochemistry (Fig. 4). Staining visualized a robust rise in TAZ expression in the epithelium, which was accompanied by enhanced nuclear localization (Fig. 4d). Importantly, CCG-1423 treatment strongly mitigated the UUO-associated increase in TAZ mRNA and protein expression (Fig. 4a–e) Together these results indicate that TAZ expression increases in the injured kidney in an MRTF-dependent manner.

**Figure 5:**
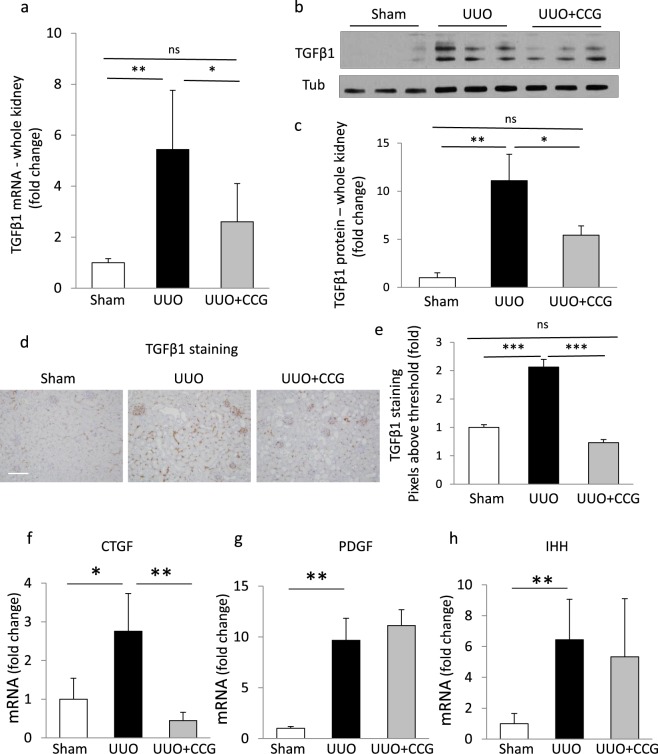
The impact of MRTF inhibition on whole kidney expression of various fibrogenic cytokines during UUO. (**a**–**e**) Renal TGFβ1 expression. Whole kidney extracts were prepared from sham, 3-day UUO and CCG 1423-treated UUO mice as in Fig. 4, and TGFβ mRNA (**a**) and protein (**b**,**c**) levels were determined by qPCR (mean ± S.D., n = 5/per group) (**a**) and western blotting (**b**) followed by densitometry (**c**), respectively. TGFβ1 protein expression was normalized to tubulin (Tub) in the same samples. The representative blot shows the result from 3 animals from each group. (Quantitation, n = 4–6/group). Note that two bands were visualized, likely corresponding to pro- and mature forms of TGFβ; both bands were quantified by densitometry. To assess localization, kidney sections were stained for TGFβ (**d**) and the staining intensities were quantified by determining the pixel percentage above background as in Fig. 4. Bar: 200 μm. The mRNA levels for other fibrogenic cytokines, namely CTGF (**f**), PDGF (**g**) and IHH (**h**) were determined from the same samples by qPCR (mean ± S.D., n = 3–5/per group). Note that UUO caused a significant increase in the expression of all investigated cytokines, of which the increases in TGFβ1 and CTGF expression were significantly attenuated by MRTF inhibition.

As TGFβ1 is the major driver of fibrogenesis, we investigated its renal expression. Early UUO resulted in substantial (5–10-fold) increase in TGFβ mRNA (Fig. 5a) and protein (Fig. 5b,c) expression. Importantly, both responses were significantly suppressed by CCG-1423 treatment (Fig. 5a–c). In agreement with previous reports^29^ immunohistochemical TGFβ1 staining showed some glomerular labeling with faint and sparse interstitial signal under basal conditions. UUO increased tubular/peritubular and interstitial TGFβ labeling (Fig. 5d). We also detected stronger glomerular staining. Overall quantitation showed that the number of positive (higher than background) pixels doubled. Importantly CCG-1423 abolished this increase (Fig. 5e). Next, we examined the expression of fibrogenic cytokines CTGF, PDGF and IHH (Fig. 5f,h). UUO caused significant increases in mRNA levels of CTGF, PDGF and IHH. CCG-1423 treatment fully abolished the rise in CTGF expression whereas PDGF and IHH were not affected. This suggests that while CTGF expression is robustly MRTF-dependent, alternative pathways are sufficient to drive expression of PDGF and IHH during UUO.

### Inhibition of MRTF suppresses tubular expression of TAZ, TGFβ1 and CTGF

**Figure 6:**
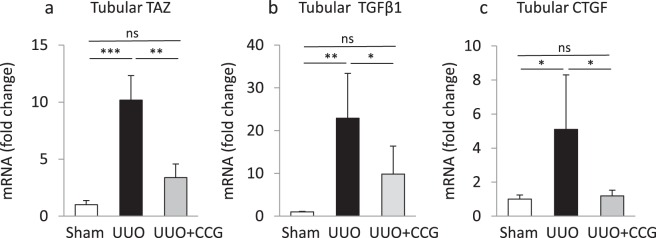
MRTF mediates *tubular* expression of TAZ, TGFβ1 and CTGF during UUO. Kidney sections from sham, UUO and CCG-1423-treated UUO mice were mounted on membrane slides and rapidly stained with H&E staining. The tubular epithelium was then isolated using laser capture microdissection. Approximately 200 tubular excisions were collected from each animal, and the obtained material was processed for qPCR to detect tubular TAZ (**a**), TGFβ1 (**b**), CTGF (**c**) mRNA levels. Note that mRNA levels of each of these genes increased significantly in the tubular epithelium during UUO, and this effect was significantly suppressed by MRTF inhibition (mean ± S.D. n = 5/per group).

Although suggestive, the above experiments do not directly show that CCG-1423 affected tubular (i.e. epithelial) transcriptional responses for TAZ and cytokines under UUO conditions. To address this crucial point, we collected tubular samples from each animal group using laser capture microdissection (LCM) and analyzed these with micro-qPCR. Using a variety of markers, we have verified the purity of the isolated tubular compartment both in sham and UUO kidneys in our previous studies^58^. LCM revealed that TAZ, TGFβ1 and CTGF mRNA significantly increased specifically in the tubules of UUO-challenged animals, and each of these responses was significantly inhibited (or in case of CTGF fully abolished) by CCG-1423 treatment (Fig. 6a–c). In agreement with the results showing a rise in MRTF protein expression by immunohistochemistry, MRTF mRNA was also significantly and specifically increased in the tubular compartment (Supplementary Fig. 2i). Moreover, the UUO-induced MRTF protein expression per se was suppressed by CCG-1423 (Supplementary Fig. 2b), suggesting that MRTF may regulate its own expression via a positive feedback loop.

In contrast, we could not detect a rise in IHH in the tubules whereas PDGF remained below the level of detectability in both control and UUO conditions (data not shown). Taken together, UUO increases renal tubular TAZ, TGFβ1 and CTGF expression in an MRTF-dependent manner.

### CCG-1423 inhibits fibrogenesis in the early phase of UUO

**Figure 7:**
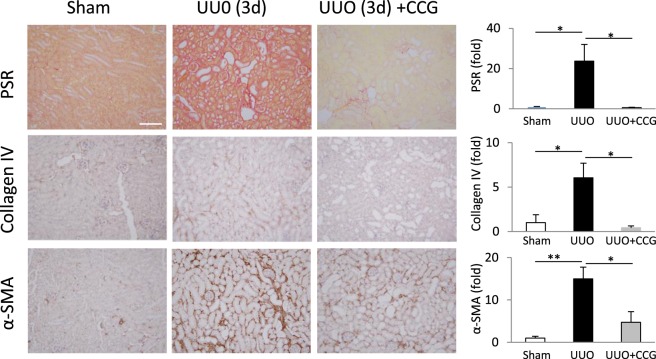
CCG-1423 treatment reduces early fibrogenesis during UUO. To assess early fibrogenesis, kidney sections were stained for fibrillary collagen using picrosirius red (PSR), and for Collagen IV and α-smooth muscle actin (α-SMA) using the respective antibodies. Quantitative immunohistochemistry revealed a significant increase in the levels of all of these fibrosis markers, an effect that was abolished or mitigated by CCG-1423 treatment; 3–5 randomly chosen fields/animal were quantified in 2 animals in each group (mean ± S.E., n = 6–10/per group). Similar results were obtained by whole-specimen scanning using a Huron Technologies TissueScope scanner and the TissueStudio analysis software (not show).

Finally, we assessed if CCG-1423 could exert overall antifibrotic effects in UUO. We therefore stained kidney samples for classical markers of ECM production (PSR or fibrillar collagens, and Collagen IV) and myofibroblast transition (α-SMA) (Fig. 7). Quantitative image analysis revealed that each marker increased in the interstitial compartment upon 3-day UUO, and that each was significantly suppressed by CCG-1423 treatment. Thus, inhibition of MRTF effectively mitigated obstructive nephropathy-induced fibrosis.

## Discussion

The central conceptual novelty of this work is the demonstration that MRTF and TAZ are key contributors to the development of the profibrotic epithelial phenotype, as these factors act as indispensable mediators of injury-induced epithelial production of fibrogenic cytokines. Furthermore, to our best knowledge our study is the first to show *in situ* activation of RhoA and increased expression/activation of MRTF in the tubular epithelium in a relevant animal model of fibrosis. While MRTF-A knockout mice have been found to be partially protected against diabetic nephropathy^50^ and genetic or pharmacological interference with MRTF mitigated fibrosis in various other organs (e.g. heart, liver, lung, and skin/systemic^49,68–71^), these effects were attributed to a direct role of MRTF in fibroblast activation (e.g. collagen and fibronectin production) and injury- or TGFβ-induced fibroblast-myofibroblast transition. Our own studies have shown a key role for MRTF in full-blown EMyT and α-SMA expression^31,32^. While these effects undoubtedly contribute to the fibrogenic activity of MRTF, our current studies suggest that MRTF also plays a central role in the initial, epithelial phase of fibrogenesis by mediating the synthesis of fibrogenic cytokines. Inhibition of MRTF by CCG-1423^60,72^ or its recently developed derivatives (e.g. CCG-203971), which have better bioavailability and efficacy^73^ represents a promising antifibrotic intervention for several reasons. First, due to the dual role of MRTF, they target both the epithelial and the mesenchymal compartments of fibrogenesis. Second, they act directly on MRTF (masking its nuclear localization signal^59^) i.e. downstream of RhoA without interfering with the non-transcriptional, widespread effects of this GTPase. Third, MRTF emerges as a master regulator of fibrogenic (partial or complete) phenotypic transitions. This conclusion is supported by our previous and current findings that (1) MRTF is a transcriptional driver of TAZ expression^35,48^; (2) TAZ levels increase during fibrogenesis in the whole kidney^48^ and, as our LCM results now show, specifically and predominantly in the tubular epithelium; and (3) increased renal/tubular TAZ expression is inhibited by CCG-1423. Moreover, elegant studies by Grande at al.^24^ have revealed that epithelial expression of Snail, another major EMT-inducing transcription factor is both necessary and sufficient to induce partial EMT accompanied by an increase in renal TGFβ1 levels and fibrosis. Interestingly MRTF was shown to drive Snail/Slug expression^46^, thus it is conceivable that MRTF is one of the key transcription factors responsible for the rise in epithelial Snail as well. In any case, the fact that three EMT-inducing transcription factors, MRTF, TAZ and Snail, are activated or overexpressed in the tubular epithelium during fibrosis gives strong credence to the notion that EMT, at least in its partial form (PEP), does occur during, and significantly contributes to the ensuing pathology.

We found that during UUO MRTF protein levels increased in the tubular epithelium and this effect, at least in part may underlie augmented MRTF activity. While the mechanisms regulating MRTF expression have not been extensively characterized, actin remodeling and TGFβ were reported to increase MRTF mRNA in an SRF-dependent manner^65,66^. Our data showing that MRTF expression per se was mitigated by CCG-1423 during UUO are consistent with an MRTF/SRF-dependent positive feedback loop for the expression of both proteins, a possibility that warrants further studies. In addition, increased RhoA signaling was also proposed to stabilize MRTF protein^67^. Taken together an initial activation of the RhoA/MRTF pathway may lead to increased MRTF expression. This, along with the consequent TAZ expression will lead to enhanced cytokine production, thereby generating a fibrogenic positive feedback loop.

Of the two main Rho-sensitive Hippo pathway effectors, in this study we have concentrated on TAZ, as UUO had a much stronger effect on this protein than YAP^48^, and TAZ but not YAP is driven by MRTF^35,48^. However, YAP is also emerging as a fibrogenic mediator^74–76^. Arguably, the relative contribution of TAZ vs. YAP might depend on the type of the fibrosis-initiating injury.

Our studies show that MRTF and TAZ are activated in mechanically challenged renal tubular cells, and both are critical for production of the most important fibrogenic cytokines. The underlying mechanisms can be both direct and indirect. Consistent with the first possibility, the CTGF promoter is known to be regulated via MRTF/SRF-driven CArG boxes, and TAZ/YAP/TEAD-driven cis-elements. Further, large-scale (ChIP-seq, microarrays) analyses have predicted SRF/MRTF-responsive CArG boxes in the promoters of TGFβ1 and PDGF-A^77^, and TAZ/YAP responsiveness for TGFβ2^78^. Nonetheless the importance of these elements under pathophysiological conditions was not assessed. Indirect mechanisms may include regulation of upstream elements of the mechanotransduction pathways. For example, MRTF is known to affect β1-integrin expression, and mechanical induction of TGFβ1 production was recently shown to be mediated by the β1 integrin/Src/Stat3 pathway^79^. It remains to be tested if MRTF and/or TAZ/YAP inhibition exert their effects partially via interfering with upstream elements of mechanosensitive routes. The MRTF-dependence of TAZ expression is one but likely not the only mechanism whereby MRTF inhibition acts, as judged by the presence of CArG boxes in the target genes and the fact that TAZ translocation is not affected by MRTF inhibition^35^. In agreement with our data, a recent study reported that deletion of the Hippo pathway adaptor Salvador1, which results in increased TAZ expression, enhanced UUO-induced fibrosis and the expression of TGFβ2 and the TGFβ II receptors *in vitro*^80^.

Little is known about the mechanoregulation of IHH. One study indicated that stretch induces IHH production in chondrocytes^81^, and a recent paper proposed that TAZ can activate IHH expression in non-alcoholic steatohepatitis^82^. Further work should clarify direct and/or indirect regulation.

It is noteworthy that the MRTF/TAZ-dependent cytokine production demonstrated herein represents a vicious circle: MRTF and TAZ activate TGFβ1 production and ECM deposition (stiffness) and both of these factors are known to activate MRTF and TAZ/YAP. Further, we have shown that the TGFβ-provoked TAZ expression is MRTF-dependent *in vitro*^48^, and a recent study published during the completion of this report reveals that epithelial overexpression of TGFβ can induce epithelial TAZ expression^83^. Such positive feedback loops are characteristic of rapid fibrogenesis, and breaking them may signify an efficient therapeutic approach. Of note, the *in vitro* and *in vivo* results overlap but are not identical; while CCG-1423 potently inhibited all of the stretch-induced cytokine responses in LLC-PK1 cells (proof of principle), CCG-1423 treatment reduced TAZ, TGFβ1 and CTGF but not PDGF or IHH in the injured kidney. In the tubular epithelium CCG-1423 suppressed the rise in tubular TAZ, TGFβ1 and CTGF, whereas at this early time point we did not see a rise in tubular IHH, and could not detect PDGF mRNA. The likely explanation is that these cytokines are driven by alternative mechanisms *in vivo* and/or that non-epithelial cells may be their major sources. The emerging picture is that activation of fibrogenic pathways both in the epithelium and in the mesenchyme contribute to fibrosis. In accordance with this notion, a recent study shows that fibroblast-specific deletion of YAP and TAZ mitigates but does not prevent fibrosis^84^.

The roles of MRTF and TAZ/YAP are by no means restricted to mechanically induced/promoted fibrosis. For example, MRTF can be activated by TGFβ1 and oxidative stress^48,85^, while the prime regulator of TAZ/YAP signaling is the Hippo pathway, which integrates humoral, metabolic, and polarity-associated inputs^37,44^. Indeed, evidence is accumulating that MRTF and TAZ/YAP play a significant role in a variety of experimentally induced fibrosis models (e.g.^48–50,53,72,80,86^) forecasting a central role for these factors in clinical disease as well.

Beside cytokine production, PEP is also associated with oxidative changes (e.g. Nox4 expression), cellular senescence and alteration in the cell cycle (G2/M block)^87^. We have shown that both MRTF and TAZ contribute to Nox4 expression^58^, and a recent study reported that MRTF upregulates Nox1 and 4 in macrophages in the context of acute kidney injury^88^. Nonetheless, it remains to be tested if MRTF and TAZ/YAP participate in the other key features of PEP.

In summary, our study reveals that MRTF and TAZ play central roles in the development of PEP, a process hallmarked by epithelial cytokine production, which in turn may play a key role in the early, triggering phase of fibrogenesis via epithelial-mesenchymal interactions.

## Materials and Methods

### Cells and reagents

LLC-PK1 (Cl 4) cells (porcine kidney tubular epithelial cells, a gift from R. C. Harris, Vanderbilt University School of Medicine, Nashville, TN), were maintained in DMEM containing 1 g/L glucose and sodium pyruvate and supplemented with 10% fetal bovine serum (FBS) and 1% penicillin/streptomycin (Invitrogen).

Reagents: TGFβ1 and Rhosin hydrochloride were from R&D Systems (Minneapolis, MN), CCG-1423, verteporfin, 3,3′-Diaminobenzidine (DAB) and picrosirius red (PSR) from Sigma Aldrich (St. Louis, MI). Antibodies were from the following sources: TAZ and TGFβ: Abcam (Cambridge, UK); MRTF-A: Cell Signaling Technologies (Danvers, MA); MRTF-B: Santa Cruz Biotech (Dallas, TX), tubulin: Sigma Aldrich (St. Louis, MI); active RhoA: New East Biosciences (King of Prussia, PA); Collagen IV: Southern Biotech (Birmingham, AL); horseradish peroxidase-coupled antibodies: Jackson ImmunoResearch Laboratories; Alexa 488 or 555-coupled antibodies: Invitrogen. 4,6-Diamidino-2-phenylindole, dihydrochloride (DAPI) was from Lonza.

All methods applied in this study were performed in accordance with the relevant guidelines and regulations of Scientific Reports and the Keenan Research Centre/St. Michael’s Hospital.

### siRNA-mediated silencing

Transfection with siRNA was performed using Lipofectamine RNAiMAX (Invitrogen). Forty-eight h post transfection cells were serum depleted for 3 h and subjected to cyclic stretch as described below. Porcine-specific siRNAs (Thermo-Fisher or Sigma-Aldrich) were against the following sequences: TAZ 5′-GGAAGAAGATCCTGCCTGA-3′; MRTF-A 5′-AACCAAGGAGCUGAAGCCAAA-3′; and MRTF-B 5′-AACGACAAACACCGTAGCAAA-3′^35^. siRNAs for MRTF-A and MRTF-B were used simultaneously at 50 nM each, and for TAZ at 50 nM. Non-related (NR) control siRNA (Applied Biosystems) was used under the same experimental conditions.

### Mechanical cell stretch

Cells were grown on six-well plates with untreated flexible bottoms (BioFlex culture plates), and serum-depleted overnight. Where indicated, 5 μM CCG-1423 or 1 μM verteporfin were added for 30 min prior to stretching. Similar drug doses were shown to be effective and non-toxic in previous *in vitro* studies by us and others^53,58,89,90^. Cells were subjected to 60 cycles/min stretch (0.5 s of stretch (10%) and 0.5 s of relaxation) for 3 h in a humidified 5% CO2 incubator at 37 °C using Flexcell 5000. Control cells were grown on the same flexible surface but not subjected to mechanical stretch.

### Unilateral Ureteral Obstruction (UUO)

The UUO procedure was described in^53,58^. Briefly, 6–8 weeks old male C57BL/6 mice (Jackson Laboratory, Bar Harbor, ME) were randomized to receive an intraperitoneal injection of 1.5 mg/kg CCG-1423 or saline prior to surgery, and daily post-surgery. This dose was chosen according to previous studies showing CCG-1423 efficacy without toxicity^72^. During surgery the left ureter was tied (UUO) or left undisturbed (sham). At 3 days post-surgery the mice were sacrificed, the left kidneys were harvested, and the tissue was either snap-frozen in liquid nitrogen (for RNA and protein), or embedded in Optimum Cutting Temperature (OCT) medium and snap-frozen (for LCM), or fixed and embedded in paraffin (for IHC). Samples were stored at −80 °C until processed. The animal care committee of the St. Michael’s Hospital approved this study.

### Real Time Quantitative PCR

Total RNA from whole kidneys or LLC-PK1 cells was purified using Total RNA Mini Kit (Geneaid, New Taipei City, Taiwan), or the RNeasy mini kit (Qiagen, Venlo, Netherlands), respectively. RNA was reverse-transcribed using iScriptTM kit (Bio-Rad), and SYBR-green-based PCR was performed and analyzed as described in^48^. Primers are listed in Table 1. Data were analyzed using iQ5 software. Melt curves confirmed the presence of one amplicon. Quantification of mRNA was done using the Livak Method.

**Table 1:** Primers used in the study.

ID	Sense	Antisense
Murine MRTF-A	5′-TTG TCC CAG CCT GGT TCT CCA-3′	5′-ATC TGC TGA AAT CTC TCC ACT CTG-3′
Murine MRTF-B	5′CCC CAG CAG TTT GTT GTT CAG CACT CTT-3′	5′- GAT GCT GGC TGT CAC TGG TTT CAT CTT G-3′
Murine TAZ	5′-GAC GAG ATG GAT ACA CCT GAA-3′	5′-GAA GGC AGT CCA GGA AAT CA-3′
Murine TGFβ1	5′-GAA GGG CCG GTT CAT GTC ATG-3′	5′-TGT GAC AGC AAA GAT AAC AAA CTC CAC-3′
Murine PDGF-B	5′-CCC ACA GTG GCT TTT CAT TT-3′	5′-GTG AAC GTA GGG GAA GTG GA-3′
Murine CTGF	5′- TTG TAA TGG CAG GCA CAG GTC -3′	5′- CGC ACA AGA ACC ACC ACT CTG -3′
Murine IHH	5′-GCT CAC CCC CAA CTA CAA TC-3′	5′-GCG GCC CTC ATA GTG TAA AG-3′
Murine GAPDH (reference gene)	5′- TGC AGT GGC AAA GTG GAG ATT-3′	5′-TTG AAT TTG CCG TGA GTG GA-3′
porcine TGFβ1	5′-CTG TGT CTG TCC ACC ATT CA-3′	5′- GGT CCA AGA TGC TCA GTA AGT-3′
porcine TGFβ2	5′-TTG ATG GCA CCT CCA CAT ATA C-3′	5′-TGT AGG AGG GCA ACA ACA TTA G-3′
Porcine CTGF	5′-GTG AAG ACA TAC CGG GCT AAG-3′,	5′-GAC ACT TGA ACT CCA CAG GAA-3′
Porcine PDGF-B	5′-CAA GTG TGA GAC GGT GGT GG-3′	5′-GCG GGG CTG AAC AAT TAG AG-3′
Porcine IHH	5′-CTT GCA GCG CTG GGT CAT-3′	5′-CGC TAT GAA GGC AAG ATC GC-3′
Porcine GAPDH (reference gene)	5′-GCA AAG TGG ACA TGG TCG CCA TCA-3′,	5′-AGC TTC CCA TTC TCA GCC TTG ACT-3′

### Laser Capture Microdissection

LCM was described in^58^. Briefly, OCT embedded mouse kidney sections were stained using Mayer’s hematoxylin and eosin Y solution (Sigma). LCM was performed using the Leica LMD laser capture microdissection system (Leica Microsystems GmbH, Wetzlar, Germany). A total of ≈200 tubular cuts were captured for each sample. Total RNA was isolated using the RNeasy micro RNA isolation kit (Qiagen) and reverse-transcribed using iScriptTM reverse transcription (Bio-Rad) and qPCR was performed using QuantiFast SYBR Green (Qiagen) with the primers listed in Table 1.

### Preparation of whole kidney lysates

Kidney samples were processed as described in^58^. Briefly, kidney samples were lysed using ice-cold RIPA buffer (50 mM Tris-HCl, pH 7.4, 150 mM NaCl, 1% Triton X-100, 1% sodium deoxycholate, 0.1% SDS, and 1 mM EDTA) supplemented with 1 mM Na_3_VO_4_, 1 mM phenylmethylsulfonyl fluoride, and Complete Mini Protease Inhibitor (Roche Applied Science, Mississauga, Ontario, Canada), homogenized and centrifuged. Supernatants were used for Western blotting.

### Western Blotting

SDS-PAGE and Western blotting were performed as in^48^. The blots were developed using enhanced chemiluminescence (GE Healthcare, Mississauga, Ontario, Canada), and the signal was captured with a ChemiDoc system (Bio-Rad). Where indicated, blots were stripped and reprobed to demonstrate equal loading or to detect levels of down-regulated proteins. Quantification was performed using the ImageLab software (Bio-Rad). Extended blots are shown in Supplementary Fig. 1.

### Immunofluorescence microscopy

Cells grown on glass coverslips or BioFlex membranes were transfected and/or treated as indicated. The staining was performed as in^35^. Images were captured using an Olympus IX81 microscope coupled to an Evolution QEi Monochrome camera using MetaMorph software or a Zeiss AxioObserver microscope, and analyzed by the Zen Blue software. Background staining was determined and subtracted using parallel staining with secondary antibody only. Representative images are shown from each condition in a minimum of three independent experiments. All image processing was done according to the Journal’s guidelines.

### Immunohistochemistry and quantification

Staining of paraffin embedded kidney sections was performed as in^53^. Histological slides were viewed by an Olympus BX50 microscope driven by the Cellsense software, and analyzed using Fiji software^91^. To quantify the amount of DAB or PSR-positive pixels in each image, color deconvolution was applied, followed by thresholding. The number of positive pixels was then expressed as fold change compared to sham condition. Additional image analysis was also performed using the Axio Scan Z1 slide scanner driven by the Zen software and the Halo V2.3 program, as described in detail in the legend to Supplementary Figure 2.

### Statistical Analysis

Data are presented as representative blots or images from at least three similar experiments or as means for the number of experiments indicated ± standard deviation (S.D.) or standard error (S.E.), as indicated. Statistical significance was determined by one-way analysis of variance (Tukey post hoc testing for parametric analysis of variance), using Prism software. *p* < 0.05 was accepted as significant. *p* < 0.05; **p < 0.01; ***p < 0.001 and ****p < 0.0001.

## Supplementary information

Supplementary Figures

## Data Availability

The datasets generated during and/or analysed during the current study are available from the corresponding author on reasonable request.
