# Supplementary material for: Profibrotic epithelial phenotype: a central role for MRTF and TAZ
Source: Sci Rep. 2019 Mar 13;9:4323. doi: 10.1038/s41598-019-40764-7 (PMC6416270; doi:10.1038/s41598-019-40764-7)
Supplement: Supplementary file 1 — Supplementary Figures [file 41598_2019_40764_MOESM1_ESM.pdf]

SUPPLEMENTARY INFORMATION

Profibrotic epithelial phenotype: a central role for MRTF and TAZ

Janne Folke Bialik, Mei Ding, Pam Speight, Qinghong Dan, Maria Zena Miranda,
Caterina Di Ciano-Oliveira, Michael M. Kofler, Ori D. Rotstein,
Stine F. Pedersen, Katalin Szászi and András Kapus

Supplementary Fig1

Figure 1a (for Fig 1)

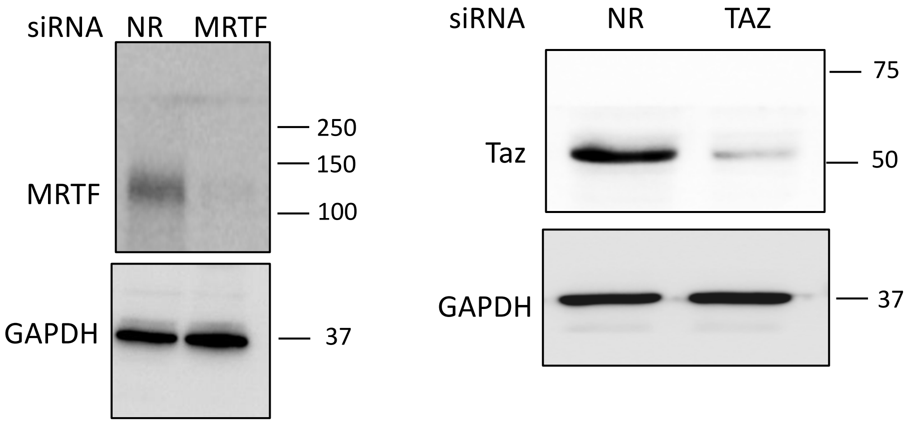

Figure 1b (For Fig 4)

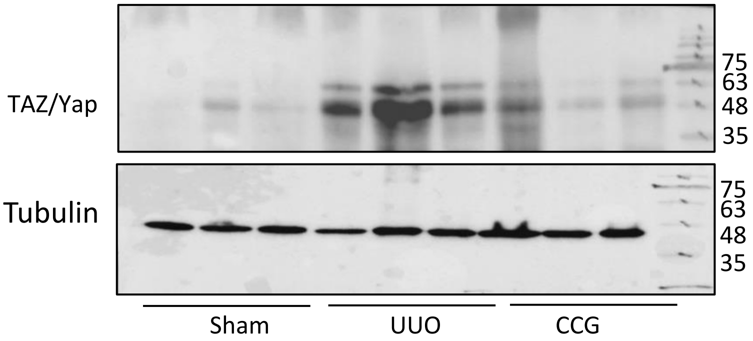

Figure 1c (for Fig 5)

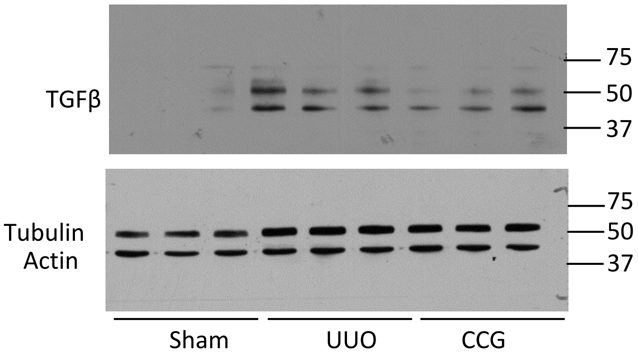

Figure 1 a-c , are the extended versions of the blots shown in the corresponding Fig 1a, Fig 4b and Fig 5b, respectively. In accordance with pervious reports (e.g. Ho et al. (2016) Int J. Mol Med 38:713-720, Chang et al (2017). Frontiers in Pharmacology 8, 1-17), the Abcam TGFβ1 antibodies. e. g. ab190503 (used in our studies) and ab179606 detect TGFβ1 in various cell types in the 45-50 kDa range, corresponding to pro-TGFβ1.

Supplementary Fig2

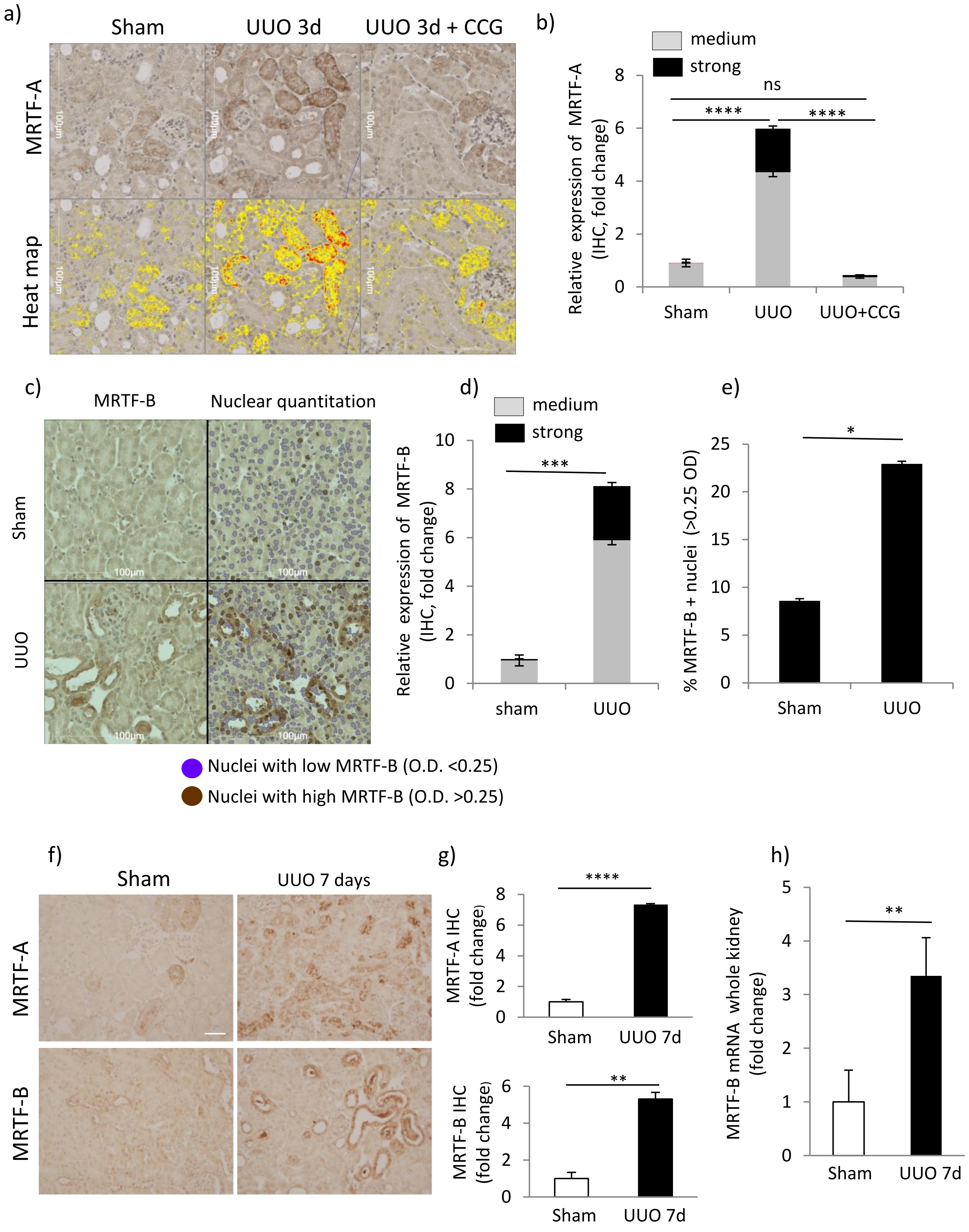

i)

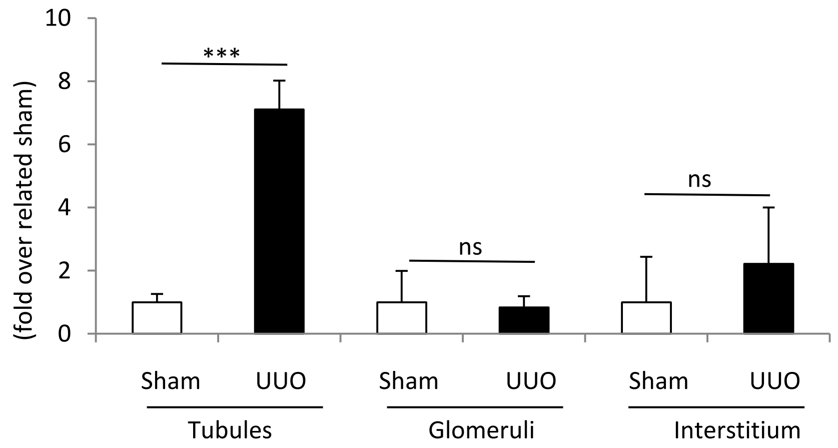

MRTF expression under sham and UUO conditions. a,b) Kidney sections from sham, 3-day UUO-challenged or 3-day UUO-challenged and CCG-treated animals were stained for MRTF-A. Quantification was performed using the Axio Scan Z1 slide scanner driven by the Zen software and analyzed by the Halo V2.3 program. From 2-3 animals in each group, 3 large (>10^6 μm^2) areas were randomly selected. After setting the threshold, positive areas were sorted into weak (optical density 0.15-0.25), medium (0.25-0.35, yellow) and strong (>0.35, red) categories (a), and their area distribution was determined (b). Data were normalized to the average area corresponding to the sum of the medium plus strong staining in the sham group. Medium (gray) and strong (black) areas are separately depicted. Significance is shown for medium staining. Only marginal strong-staining areas were present in sham and CCG+UUO. c, d, e) MRTF-B staining was quantified as in a and b). The right panels in c show automated nuclear identification, defined by hematoxylin staining. Nuclei with low (O.D. < 0.25) and high (O.D. >0.25) staining for MRTF are shown using blue and brown masks, respectively. d) Total MRTF-B expression. Positive areas exhibiting weak, medium and strong staining were identified and quantified as in b. and normalized to the average area corresponding to the sum of the medium plus strong staining in the sham group. Medium (gray) and strong (black) areas are separately depicted. Significance is shown for medium staining. e) Percentage of nuclei showing medium/strong MRTF-B staining (O.D. >0.25). For b, d and e, mean ± S.E.M. is shown (n=6-9). f,g) Immunohistochemical staining was performed for MRTF-A and B after sham and 7-day UUO surgery (f) and quantified (g) as described in the Methods. h) MRTF-B mRNA was measured in whole kidney extracts after sham and 7-day UUO surgery (n=3). i) MRTF-A mRNA expression was measured by qPCR in various kidney compartments isolated by laser capture microdissection from sham and 7-day UUO-treated animals (n=3, mean ± S.D.). Significance was determined by t-test comparing expression in the same compartment (n=3, mean ± S.D., ns, non-significant).
